# Prognostic Value of the Largest Lesion Size for Progression-Free Survival in Patients with NET Undergoing Salvage PRRT with [^177^Lu]Lu-DOTATOC

**DOI:** 10.3390/cancers14071768

**Published:** 2022-03-31

**Authors:** Markus Galler, Julian M. M. Rogasch, Kai Huang, Henning Jann, Kristina Plehm, Christoph Wetz, Holger Amthauer

**Affiliations:** 1 Department of Nuclear Medicine, Charité—Universitätsmedizin Berlin, Corporate Member of Freie Universität Berlin and Humboldt—Universität zu Berlin, Augustenburger Platz 1, 13353 Berlin, Germany; julian.rogasch@charite.de (J.M.M.R.); kai.huang@charite.de (K.H.); christoph.wetz@charite.de (C.W.); holger.amthauer@charite.de (H.A.); 2 Berlin Institute of Health at Charité—Universitätsmedizin Berlin, Charitéplatz 1, 10117 Berlin, Germany; 3 Department of Hepatology and Gastroenterology, Charité—Universitätsmedizin Berlin, Corporate Member of Freie Universität Berlin and Humboldt—Universität zu Berlin, Augustenburger Platz 1, 13353 Berlin, Germany; henning.jann@charite.de (H.J.); kristina.plehm@charite.de (K.P.)

**Keywords:** PRRT, salvage PRRT, neuroendocrine tumor, De Ritis ratio, lesion size, DOTATOC

## Abstract

**Simple Summary:**

Peptide receptor radionuclide therapy (PRRT) using radionuclide-labeled somatostatin analogues is based on the overexpression of somatostatin receptors on neuroendocrine tumors and is shown to have a good safety profile and efficacy in different types of metastatic neuroendocrine tumors. As this therapy is usually not curative, most patients experience disease progression after initial PRRT. In these cases, retreatment with PRRT, also called salvage PRRT, can be a treatment option, but little is known about the efficacy and possible risk factors. In this retrospective study that included 32 patients, we found that the size of the largest lesion is a significant predictor of disease progression after salvage PRRT. This risk factor is easy to obtain and can help identify patients who may benefit from intensified follow-up strategies.

**Abstract:**

(1) Background: retreatment with radionuclide-labeled somatostatin analogues following disease progression after initial treatment cycles is often referred to as salvage peptide receptor radionuclide therapy (salvage PRRT). Salvage PRRT is shown to have a favorable safety profile in patients with metastatic neuroendocrine tumors (NETs), but numerous questions about the efficacy and prognostic or predictive factors remain to be answered. The purpose of this study was to evaluate two parameters that have shown prognostic significance in progression-free survival (PFS) in initial PRRT treatment, namely the size of the largest lesion (LLS) and the De Ritis ratio (aspartate aminotransferase (AST)/alanine aminotransferase (ALT)), as prognostic factors in the context of salvage PRRT. In addition, the PFS after initial PRRT was evaluated as a predictor of the PFS following salvage PRRT. (2) Methods: retrospective, monocentric analysis in 32 patients with NETs (gastroenteropancreatic, 23; unknown primary, 7; kidney, 1; lung, 1) and progression after initial PRRT undergoing retreatment with [^177^Lu]Lu-DOTATOC. The prognostic values of LLS, the De Ritis ratio, and PFS after initial treatment cycles regarding PFS following salvage PRRT were evaluated with univariable and multivariable Cox regression. PFS was defined as the time from treatment start until tumor progression according to RECIST 1.1 criteria, death from any cause or start of a new treatment due to progression of cancer-related symptoms (namely carcinoid syndrome). (3) Results: progression after salvage PRRT was observed in 29 of 32 patients with median PFS of 10.8 months (95% confidence interval (CI), 8.0–15.9 months). A higher LLS (hazard ratio (HR): 1.03; *p* = 0.002) and a higher De Ritis ratio (HR: 2.64; *p* = 0.047) were associated with shorter PFS after salvage PRRT in univariable Cox regression. PFS after initial PRRT was not associated with PFS following salvage PRRT. In multivariable Cox regression, only LLS remained a significant predictor. (4) Conclusions: the size of the largest lesion is easy to obtain and might help identify patients at risk of early disease progression after salvage PRRT. Validation is required.

## 1. Introduction

Neuroendocrine neoplasms (NENs) comprise a heterogeneous group of neoplasms originating from the neuroendocrine system and are usually classified according to the primary site and the grade of differentiation [1]. The spectrum of NEN ranges from well-differentiated, slowly growing neuroendocrine tumors (NETs) with favorable prognoses to poorly differentiated neuroendocrine carcinoma(s) (NEC) with a high risk of metastatic disease and poor prognosis [2]. NETs are graded as G1–3 based on the mitotic count and the proliferation marker Ki-67 [3]. The most common primary sites of NETs are the gastrointestinal tract and the pancreas [4]. These NETs are referred to as gastroenteropancreatic NETs (GEP-NET). The overexpression of somatostatin receptors (SSTR), which is frequently observed in NENs, forms the basis for therapy with radionuclide-labeled somatostatin analogues [5]. Peptide receptor radionuclide therapy (PRRT) is usually performed with the β-emitting ^177^Lu in four treatment cycles and with a treatment interval of 2–3 months, demonstrating high efficacy and low toxicity in patients with metastatic midgut-NET [6]. The presence of large lesions and higher De Ritis ratios (aspartate aminotransferase (AST)/alanine aminotransferase (ALT)) predicted shorter progression-free survival (PFS) after PRRT [7,8].

Salvage PRRT with ^177^Lu-labeled somatostatin analogues following disease progression after initial PRRT cycles is a subject of ongoing research and little is known about its efficacy and prognostic factors [9]. Considering the challenges in therapeutic options and clear guideline recommendations, prognostic biomarkers and morphological/functional imaging paving the way to salvage PRRT are unmet needs. Several retrospective studies confirm good safety profiles and favorable PFS after salvage PRRT, although most analyses miss comparisons with control groups [10,11,12,13,14,15,16]. Van der Zwan et al. reported a significantly longer overall survival in patients with bronchial NET, GEP-NET, or midgut-NET undergoing salvage PRRT than in a nonrandomized control group [12]. A high burden of liver metastases and short PFS after initial therapy cycles were found to correlate with a shorter PFS after salvage therapy [11,13,17].

The purpose of this retrospective, single center study was to evaluate the largest lesion size and the De Ritis ratio, which have shown prognostic significance in initial PRRT cycles, as predictors of PFS after salvage PRRT. In addition, PFS after initial therapy cycles was evaluated as a prognostic marker for PFS after salvage PRRT in our cohort. The results should provide useful information for the risk stratification and improvement of follow-up strategies in patients with NET undergoing salvage PRRT.

## 2. Materials and Methods

Thirty-two patients with NET undergoing salvage PRRT with [^177^Lu]Lu-DOTATOC between July 2013 and November 2020 were included in this retrospective, monocentric analysis. All patients fulfilled the following inclusion criteria: (1) histologically confirmed neuroendocrine tumor; (2) progression according to RECIST 1.1 criteria after initial PRRT; (3) persistent SSTR expression confirmed by SSTR-specific scintigraphy or positron emission tomography before salvage PRRT; (4) presence of baseline imaging performed with contrast-enhanced (CE) computed tomography (CT) or magnetic resonance imaging (MRI) < 3 months before salvage PRRT; and (5) no myocardial infarction < 1 month before salvage PRRT possibly influencing the De Ritis ratio. Patients were included in the analysis irrespective of the following criteria: (A) radionuclide used for the initial PRRT (^90^Y or ^177^Lu); (B) the presence/absence and type of additional therapies following progression after initial PRRT, provided that a progression—according to RECIST 1.1—occurred after the additional therapy and before salvage PRRT; (C) ongoing medication with somatostatin analogues. Initial PRRT was performed with a standardized number of cycles as described in detail in Section 3. Treatment recommendations for salvage PRRT were discussed and confirmed by a multidisciplinary NET conference. Salvage PRRT was performed with two treatment cycles, and an additional third cycle could be administered in patients with good tolerance and evidence of response. Planned activity per cycle was 6–7 GBq and activity was reduced in patients with risk factors for toxicity, e.g., chronic renal insufficiency.

The largest lesion size (LLS) prior to salvage PRRT was defined as the maximum diameter of the largest tumor lesion in the transaxial plane of the baseline CE-CT or MRI, irrespective of central tumor necrosis. Lytic or sclerotic bone metastases in CE-CT or MRI were also considered as measurable lesions. If the largest tumor lesion was located in the liver, the disease was defined as predominantly hepatic. AST and ALT were measured in the pretherapeutic blood serum or heparin plasma one day before the first cycle of salvage PRRT. A follow-up with CE-CT or MRI was performed 3–6 months after the retreatment conclusion and, subsequently, every 6–12 months. PFS was defined as the time from PRRT start until (1) tumor progression according to RECIST 1.1 criteria; (2) death from any cause; or (3) the start of a new treatment due to progression of cancer-related symptoms (namely carcinoid syndrome). Toxicities to organs at risk were assessed according to the CTCAE v.4.0 classification.

A swimmer plot was created to illustrate the clinical course of all study patients. A statistical analysis was performed using R Statistical Software, version 4.0.3 [18]. *p* values of less than 0.05 were considered statistically significant. The Kaplan–Meier method was used to estimate median PFS and to analyze overall survival. The prognostic values of the continuous variables (LLS, De Ritis ratio, and PFS after initial PRRT) regarding PFS following retreatment were analyzed with univariable and multivariable Cox regression. The assumption of constant hazard ratios (HR) over time was analyzed by calculating the Schoenfeld residuals and was fulfilled by every variable. For visualization purposes and to account for nonlinear effects, penalized spline-based hazard ratio curves for the LLS and the De Ritis ratio were created with the R package smoothHR [19]. An optimal cut-off value for LLS was identified based on the minimal *p*-value in the log-rank test [20]. The Wilcoxon rank-sum test was used to compare predominantly hepatic diseases and non-predominantly hepatic diseases with respect to the De Ritis ratio. The correlation between the largest lesion size and the De Ritis ratio was calculated by the Pearson correlation. In addition, the Wilcoxon rank-sum test was used to compare patients with type 2 diabetes and without type 2 diabetes, with respect to the De Ritis ratio.

## 3. Results

**Figure 1:**
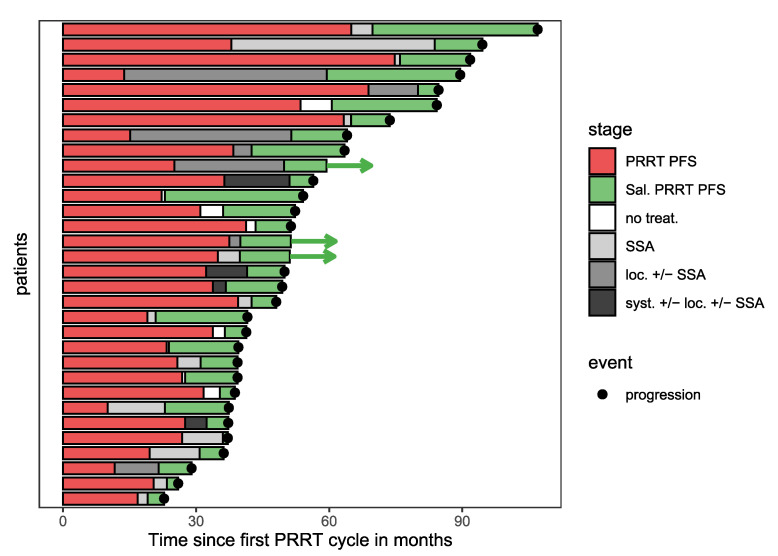
Swimmer plot illustrating clinical course of study patients. The red bars represent the PFS after initial PRRT, the green bars the PFS after salvage PRRT. The time between progression after the initial PRRT and the start of salvage PRRT is illustrated by bars with grayscale colors indicating the intensity of additional therapies in this period (white: no treatment; light grey: somatostatin analogues; dark grey: local therapies ± somatostatin analogues; black: systemic treatments other than somatostatin analogues (chemotherapy, tyrosine kinase inhibitors, mTOR inhibitors) ± local therapies ± somatostatin analogues). Arrows indicate censored patients regarding PFS.

Patient characteristics are summarized in Table 1. Most patients had GEP-NETs, followed by unknown primary (CUP). Most common metastatic sites were the liver and lymph nodes. Initial PRRT was performed with a standardized number of cycles, which was changed from three to four cycles. One patient deviated from this standardized number and underwent five initial PRRT cycles. The clinical course of all subjects is illustrated in Figure 1. The median PFS after initial PRRT was 31 months. A total of 24 (75%) patients had predominantly hepatic disease prior to salvage PRRT. Retreatment was performed with a median of 2 (range: 1–3) cycles. In 4 of 32 patients, an early symptomatic disease progression confirmed by CE-CT or MRI occurred before a scheduled second salvage PRRT cycle. The median cumulative activity of salvage PRRT was 12.0 (range: 5.4–22.2) GBq and the median activity per cycle was 6.0 (range: 4.0–7.6) GBq. The substantially reduced activity of 4.0 GBq was administered in one patient with chronic kidney disease after nephrectomy. The cycle interval was 2–3 months. One patient with treatment-induced grade 3 anemia and six patients with grade 1 thrombocytopenia were observed during salvage PRRT.

**Figure 2:**
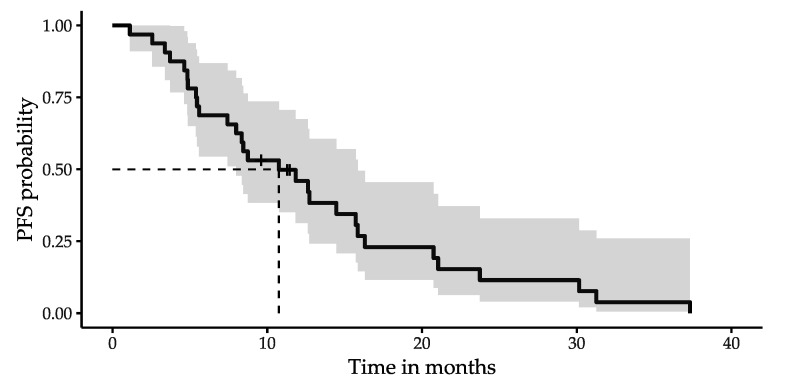
Kaplan–Meier curve of PFS after salvage PRRT with 95% CI. Censored subjects are indicated as tick marks. The median PFS equals 10.8 months (95% CI: 8.0–15.9 months).

Progression after salvage PRRT was observed in 29 of 32 patients (91%; progression according to RECIST 1.1, 25; death from any cause, 3; start of a new treatment due to progression of cancer-related symptoms, 1). The median PFS after salvage PRRT was 10.8 months (95% confidence interval (CI): 8.0–15.9 months) as illustrated by the Kaplan–Meier curve in Figure 2. The median follow-up time in patients without progression was 11 (range: 10–11) months.

**Figure 3:**
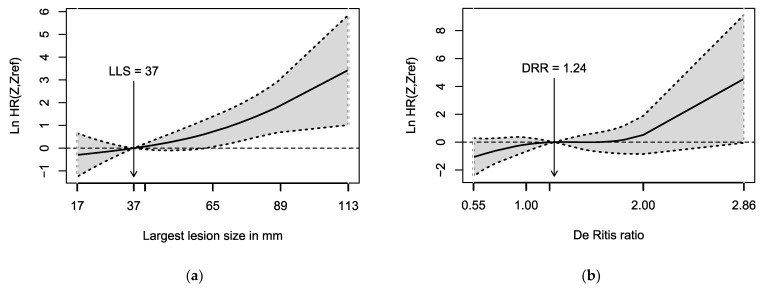
Penalized spline-based log hazard ratio curves for identification of the effects of (**a**) the largest lesion size (LLS) and (**b**) the De Ritis ratio (DRR) on progression-free survival after salvage PRRT. The solid line presents the log hazard ratio with respect to the median value, while the dotted line indicates the corresponding 95% confidence limits. At the specified reference value (median), the log hazard ratio equals zero (corresponding to a hazard ratio of one) by definition. The degrees of freedom of the penalized splines were calculated according to the Akaike information criterion [19].

In univariable Cox regression, a higher De Ritis ratio (hazard ratio (HR): 2.64, 95% CI: 1.01–6.87; *p* = 0.047) and larger LLS (HR: 1.03, 95% CI: 1.01–1.05; *p* = 0.002) were associated with a shorter PFS after retreatment. The PFS after initial therapy cycles did not predict the PFS following retreatment (HR: 0.99, 95% CI: 0.97–1.01; *p* = 0.4). The LLS was also a significant predictor of PFS in the multivariable Cox regression (HR: 1.03, 95% CI: 1.01–1.05; *p* = 0.004; Table 2). The De Ritis ratio and the PFS after initial therapy cycles were not significant predictors in the multivariable Cox regression (each *p* > 0.05). Spline-based hazard ratio curves, shown in Figure 3, confirm an increasing HR with rising LLS. For the De Ritis ratio, the hazard ratio curve also increases with higher values, but the wide confidence interval includes the zero-effect line. The hazard ratio curves do not indicate large deviations from the assumption of the log-linear relationship. The optimal cut-off value for LLS was 60.5 mm (median PFS: 5.4 (95% CI: 3.9–6.9) months versus 15.7 (95% CI: 11.6–19.8) months, *p* < 0.001).

Patients with predominantly hepatic disease did not have significantly higher De Ritis ratios than patients with the largest lesions located in other organs (the median De Ritis ratio of patients with predominantly hepatic disease was 1.32 compared to 1.19 of patients without predominantly hepatic disease, Wilcoxon rank-sum test: *p* = 0.37). The correlation analysis between the largest lesion size and the De Ritis showed a weak positive correlation (Pearson correlation coefficient = 0.22). Patients with type 2 diabetes did not have significantly higher De Ritis ratios than patients without type 2 diabetes (median De Ritis ratio: 1.80 versus 1.23, Wilcoxon rank-sum test: *p* = 0.053). Death was observed in 15 of 32 patients during follow-up, resulting in an estimated rate of overall survival at 24 months of 66% (95% CI: 51–86%; median follow-up time in patients without death: 44 months).

## 4. Discussion

The median PFS after salvage PRRT of approximately 11 months observed in this analysis is similar to the median PFS of 13 months as reported in the meta-analysis of Strosberg et al. for salvage PRRT [21]. No high-grade toxicity was observed in our cohort, except for one patient with grade 3 anemia. This confirms the relatively good safety profile of salvage PRRT reported in previous studies.

It is known that the therapeutic effect of β-emitting radionuclides depends, among other factors, on the lesion size and the path length of the β-particles [22]. For instance, ^177^Lu is more suitable for small tumor lesions due to its relatively short path length. This has been confirmed in animal studies, which analyzed the effects of ^177^Lu- and ^90^Y-labeled somatostatin-analogues on tumor lesions with different sizes [23,24]. These previous results suggest that the negative impacts of large tumor lesions on the PFS observed in our study could be related to the limited therapeutic effects of ^177^Lu in large lesions. Similar results were obtained in the NETTER-1 study, where the absence of a large lesion (>3 cm) was associated with improved PFS in patients with midgut-NET undergoing PRRT with [^177^Lu]Lu-DOTATATE [7]. In the control arm of the NETTER-1 study, the presence or absence of a large lesion did not impact the PFS. These results indicate that large lesions are possibly not only a sign of an advanced disease stage with poor prognosis and earlier disease progression, but also, as mentioned before, a potential predictive factor for salvage PRRT. A control group to substantiate this hypothesis is missing in this study. An additional effect that could impair the efficacy in large tumor lesions is based on a reduced perfusion of the tumor lesion due to central necrosis leading to a lower uptake of [^177^Lu]Lu-DOTATOC. Histopathologic studies underlined that necrosis correlates with a significant reduction in locoregional blood flow [25]. For future studies, it might be worth considering a dosimetry-based choice of activity to account for different tumor loads and lesion sizes, which could improve the efficacy of salvage PRRT. Apart from the importance for PRRT, in previous studies, the lesion size was found to correlate with the differentiation grade in pancreatic NETs [26], with prognosis in rectal NETs [27], and with response to ^90^Y-radioembolization of hepatic metastasis in NETs [28].

While a higher De Ritis ratio was associated with a shorter PFS after salvage PRRT in the univariable Cox regression, it did not show a significant association with PFS in multivariable Cox regression. This could be related to the weak positive correlation between the largest lesion size and the De Ritis ratio and low statistical power due to a small sample size. The De Ritis ratio is defined as the ratio between the serum level of aspartate transaminase (AST) and alanine transaminase (ALT). AST exists as two isoenzymes, one located in the cytoplasm, the other located in mitochondria. AST is found in various tissues including the liver, muscle, heart, and kidney [29]. ALT is mainly present in the cytoplasm of hepatocytes and, to a smaller extent, in other tissues. Elevations of AST and ALT in the serum can be caused by cell damage with plasma membrane disruption [30]. Depending on the pathology, the relative elevations of AST and ALT can differ and lead to an increased or reduced De Ritis ratio [29]. An extensive disintegration of hepatocytes can lead to a substantial rise of AST in the serum due to the release of mitochondrial AST after additional disruption of the mitochondrial membrane [31]. Therefore, a possible explanation for a relationship between a high De Ritis ratio and shorter PFS after salvage PRRT could be that high De Ritis ratios indicate advanced, aggressive hepatic involvement. However, patients with predominantly hepatic diseases did not have significantly higher De Ritis ratios in our study. Another possible effect that could lead to an elevated De Ritis ratio in tumor patients is based on the essential role of glutamine metabolism in cancer cells [32]. Glutamine supplies nitrogen and carbon for biosynthetic reactions, and AST is known to be important for glutamine metabolism in tumor cells [33,34,35]. Theoretically, an increased AST expression in tumor cells could lead to an elevated serum level of AST and indicate a high tumor proliferation. Prognostic significance of the De Ritis ratio has been shown for patients with NET undergoing initial PRRT cycles [8]. Moreover, a prognostic role of the De Ritis ratio has been observed in various other tumor entities, including urothelial carcinoma, pancreatic cancer, and prostate cancer [36,37,38]. It is worth noting that, apart from an acute myocardial infarction, which is an exclusion criterion, the De Ritis ratio could be affected by various other diseases, e.g., type 2 diabetes [39], acute ischemic stroke [40], or occlusive peripheral arterial disease [41]. However, none of the patients in this analysis had an acute ischemic stroke or occlusive peripheral arterial disease, and seven patients with type 2 diabetes did not have significantly higher De Ritis ratios compared to the other patients.

Our analysis did not reveal a significant association between PFS after initial PRRT cycles and PFS after salvage PRRT, whereas such an association was reported in previous studies by Sabet et al. (33 patients) and van Essen et al. (33 patients) [11,17]. These different observations may be the results of different characteristics in patients who had been referred to salvage PRRT or differences in additional therapies after initial treatment cycles. Furthermore, statistical methods to analyze the association between initial PFS and salvage PFS were considerably different, which complicates a direct comparison (Sabet et al.: correlation analysis; van Essen et al.: t test; present analysis: Cox regression). From a theoretical point of view, one could argue that initial PRRT cycles or additional therapies lead to interindividual changes in tumor cell biology, such as alterations in the aggressiveness or the expression of somatostatin receptors. These in turn could change the efficacy of salvage PRRT or the tumor dynamics. On the other hand, the interindividual changes in tumor cell biology between initial PRRT cycles and salvage PRRT could be small, which could lead to longer PFS after salvage PRRT in patients who previously benefited from PRRT. Further studies with larger sample sizes are needed to clarify this aspect.

This study is limited by its retrospective nature and variations in the number of salvage PRRT cycles, according to the routine clinical care of the patients. It included a relatively small number of patients with multiple primary sites and, therefore, did not account for the characteristics of different primary locations. The cohort of this analysis could be biased towards patients with good responses after initial PRRT, as these patients are primarily considered for salvage PRRT by multidisciplinary consensus. However, PFS after initial PRRT in this cohort (median: 31 months) does not differ substantially from previously reported PFS after PRRT, e.g., 34 months (95% CI: 26–42 months) in pancreatic NETs G1–2 [42] or compared to the estimated rate of progression-free survival at month 20 of 65.2% (95% CI: 50.0–76.8) in the NETTER-1 study [6].

The scope of our analysis was restricted to prognostic markers in salvage PRRT. The absence of a control group in our analysis does not allow to draw conclusions about the efficacy of salvage PRRT and the distinction between prognostic and predictive significance of the analyzed variables. Despite these limitations, the results obtained in this study provide insight into important variables for salvage PRRT and can form a basis for prospective studies with control groups.

## 5. Conclusions

The size of the largest tumor lesion was a risk factor for early disease progression in patients with NETs undergoing salvage PRRT in the current retrospective analysis. This risk factor is simple to assess and might help identify patients who may benefit from intensified follow-up strategies. In consideration of previous studies, we hypothesize that the lesion size could have a predictive significance, meaning that efficacy of salvage PRRT could possibly be reduced in patients with large tumor lesions. A high De Ritis ratio could be an additional risk factor for early disease progression after salvage PRRT, but further studies with larger sample sizes may be needed to clarify these aspects.

## Figures and Tables

**Table 1:** Patient characteristics.

Variable	*n* (%) or Median (Range)
Patient count	32
Sex	
Men	19 (59%)
Women	13 (41%)
Age in years	67 (32–81)
Charlson comorbidity index	3 (0–10)
Type 2 diabetes	7 (22%)
Occlusive peripheral arterial disease	0 (0%)
Ischemic stroke <1 month before salvage PRRT	0 (0%)
Primary location	
Gastrointestinal	18 (57%)
Pancreas	5 (15%)
Lungs	1 (3%)
Kidney	1 (3%)
Unknown (CUP)	7 (22%)
Grade	
G1	6 (19%)
G2	25 (78%)
G3	1 (3%)
Functional NET	12 (38%)
Add. treatment before/after initial PRRT	
Operative treatment	20 (63%)/2 (6%)
Somatostatin analogues	19 (59%)/21 (66%)
Chemotherapy	8 (25%)/1 (3%)
mTOR inhibitor	5 (16%)/3 (9%)
Tyrosine kinase inhibitor	1 (3%)/0 (0%)
Radiation therapy	3 (9%)/4 (13%)
Local ablative therapy	4 (13%)/3 (9%)
Number of initial PRRT cycles	3 (3–5)
Initial PRRT performed with ^177^Lu	30 (94%)
Cumulative activity during initial PRRT (GBq)	21.1 (12.0–30.3)
PFS after initial PRRT in months	31 (10–75)
Time between end of initial PRRT and salvage PRRT in months	30 (11–70)
Metastatic sites before retreatment	
Liver	31 (97%)
Lymph node	22 (69%)
Bone	16 (50%)
Peritoneum	7 (22%)
Lungs	3 (9%)
Others (muscle, ovary, spleen)	3 (9%)
Largest lesion size in mm	37.5 (17–113)
Largest lesion position	
Liver	24 (75%)
Lymph nodes	5 (16%)
Bone	1 (3%)
Others	2 (6%)
AST in U/L	26.5 (15–136)
ALT in U/L	21 (10–91)
De Ritis ratio	1.24 (0.55–2.86)
Number of retreatment cycles	2 (1–3)

**Table 2:** Univariable and multivariable Cox regression for PFS. All variables were included as continuous variables.

Univariable Cox Regression			
**Variable**	**Hazard Ratio**	**95% CI**	***p*-Value**
Largest lesion size (LLS) in mm	1.03	1.01–1.05	0.002
De Ritis ratio	2.64	1.01–6.87	0.047
PFS after initial PRRT in months	0.99	0.97–1.01	0.400
**Multivariable Cox Regression**			
**Variable**	**Hazard Ratio**	**95% CI**	***p*-Value**
Largest lesion size (LLS) in mm	1.03	1.01–1.05	0.004
De Ritis ratio	2.46	0.94–6.43	0.066
PFS after initial PRRT in months	0.99	0.97–1.02	0.578

## Data Availability

The data presented in this study are available upon request from the corresponding author.
